# Spinal growth velocity versus height velocity in predicting curve progression in peri-pubertal girls with idiopathic scoliosis

**DOI:** 10.1186/s12891-016-1221-6

**Published:** 2016-08-26

**Authors:** Benlong Shi, Saihu Mao, Zhen Liu, Xu Sun, Zezhang Zhu, Feng Zhu, Jack C. Y. Cheng, Yong Qiu

**Affiliations:** 1 Spine Surgery, the Affiliated Drum Tower Hospital of Nanjing University Medical School, Zhongshan Road No. 321, Nanjing, 210008 China; 2 Department of Orthopaedics and Traumatology, Chinese University of Hong Kong, Hong Kong, China; 3 Joint Scoliosis Research Center of the Chinese University of Hong Kong & Nanjing University, Nanjing, China

**Keywords:** Idiopathic scoliosis, Spinal growth velocity, Height velocity, Angle velocity, Curve progression

## Abstract

**Background:**

Height velocity (HV) is traditionally used to monitor the residual growth potential in idiopathic scoliosis (IS). The temporal timing of rapid increase in standing height often does not match exactly that of the increase in spine height. The purposes of this study were to analyze the correlation between change of angle velocity (AV) vs the changes of spinal growth velocity (SGV) and HV, and the associated predictive value on curve progression in IS.

**Methods:**

Pre-pubertal IS girls with single curve receiving standardized bracing treatment followed longitudinally with documented curve progression >5° were retrospectively reviewed. The age, standing height, Cobb angle (main curve), spinal length, Risser sign, HV, SGV and AV at each visit were measured and calculated. The visit with the highest AV value of each patient was selected for the final analysis and correlated with the corresponding peak height velocity (PHV) and peak spinal growth velocity (PSGV).

**Results:**

Sixty-two IS girls were reviewed. Chi-square test revealed PSGV contributed more to the highest AV than PHV (*P* = 0.001). Pearson correlation analysis demonstrated that AV was correlated with SGV (*r* = 0.454, *P* < 0.001) and HV (*r* = 0.280, *P* = 0.027). Multiple linear regression analysis showed that high AV was better predicted by higher SGV (B = 0.321, *P* = 0.007) rather than higher HV (B = 0.259, *P* = 0.362) (*R* = 0.467).

**Conclusions:**

Variations of spinal growth velocity exerted more direct influence over changes in angle velocity as compared with height velocity. High spinal growth velocity predisposed to more rapid curve progression in patients with idiopathic scoliosis.

## Background

Idiopathic scoliosis (IS) is prevalent among adolescents during the pubertal spurt and in the severe cases, may lead to significant morbidities [1, 2]. The risk of curve progression was found to correlate significantly with period of rapid skeletal linear growth and in particular the time relative to the peak height velocity (PHV) [3, 4]. The height velocity (HV) has thus provided useful reference information of the residual growth potential and could inform the treatment strategy clinically [5, 6]. The calculation of HV, however, would require serial longitudinal data of standing height, comprising the summation of the sitting height and subischial height. Busscher et al. [7] reported that growth occurs in multi-dimensions, at different rates in different parts of the body, and noted the existence of a distal-to-proximal growth gradient in adolescents. The temporal timing of the rapid increase of standing height during pubertal spurt often does not match exactly that of the increase in spine height. The alternative of using sitting height to avoid the influence of asynchronous growth in the lower limbs is also limited by the lack of reliable methods in assessing the true loss of spinal length resulting from curve progression [8].

We hypothesized that direct monitoring of spinal growth velocity (SGV), free of the two aforementioned limitations, might have higher predictive value of curve progression during the rapid pubertal growth as compared to the conventional HV in IS. Current information concerning the velocity of spinal growth and its relationship with curve progression is, however, inadequate. The aims of this study were to analyze the correlation between change of angle velocity vs the changes in growth velocity of standing height and spine height, and the associated predictive value on curve progression in idiopathic scoliosis.

## Methods

### Subjects

This retrospective study was approved by the Ethics Committee of Drum Tower Hospital of Nanjing University Medical School. IS patients treated with standardized bracing programme from January 2008 to September 2010 with complete set of serial anthropometric and radiographic measurements were reviewed. The indication for bracing treatment were: (1) initial chronologic age <14 years and menarche age <1 year; (2) Risser sign between 0 and 2; (3) initial major curve magnitude between 20 and 40°; and (4) no prior treatment history. Enrolment into the current study was limited to: (1) IS girls with single thoracic or thoracolumbar/lumbar curve treated with standard Milwaukee or Boston brace and followed up until brace weaning [9]; (2) menarche age less than 6 months and Risser 0 at the initiation of bracing; (3) compliance of the bracing treatment of more than 75 %; and (4) curve progression of more than 5° at final follow-up. The exclusion criteria were: (1) patients with previous spinal surgery; (2) patients with any signs of growth abnormalities (such as a lower extremity growth arrest or deficiency), neurological abnormality, skeletal dysplasia or dwarfism.

### Anthropometric and radiographic measurements

**Fig. 1:**
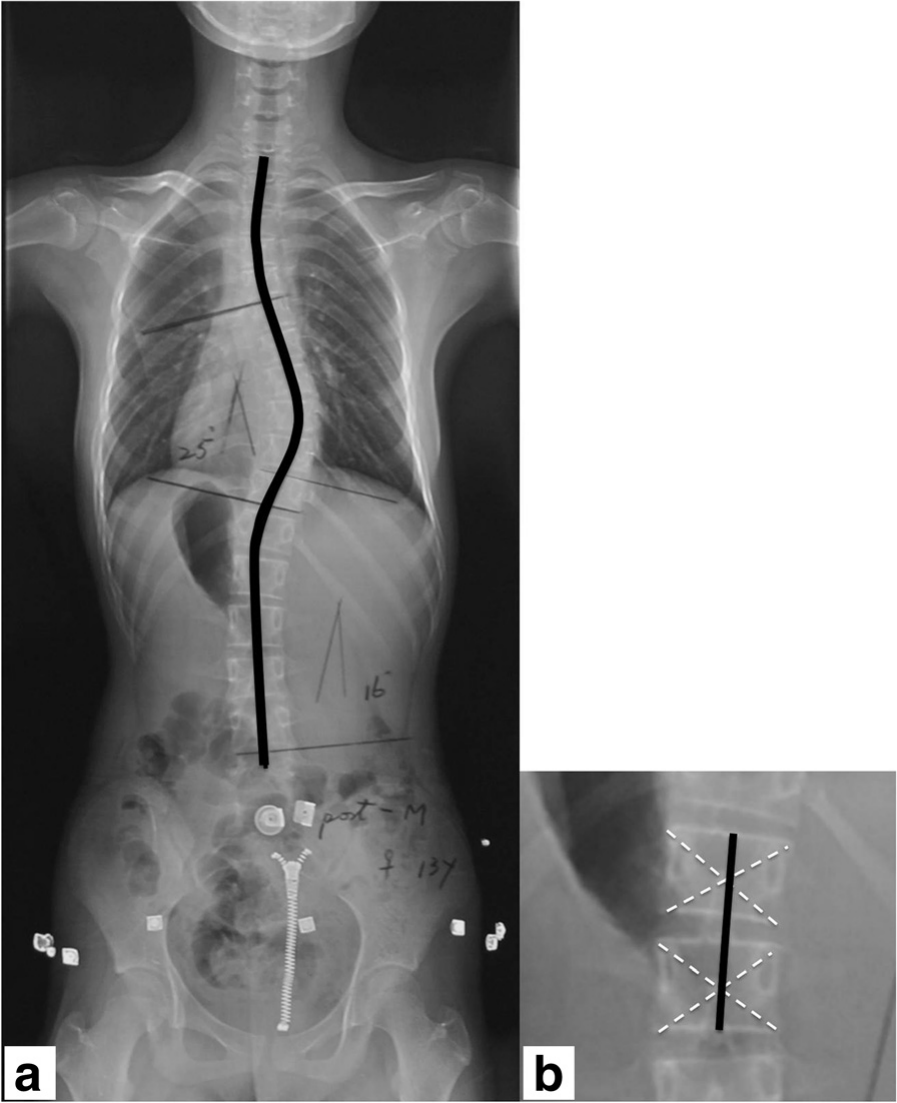
Spinal length was measured by the line through the midpoints of superior endplate, diagonal intersection of each vertebra, midpoints of inferior endplate and discs from the superior endplate of T1 to the inferior endplate of L5. The major and minor curves were (**a**) 25° and (**b**) 16°, respectively

For the anthropometric data, standing height was measured in centimeters by a senior resident using a wall-mounted ruler with a perpendicular slide at each visit in standing position, looking straight ahead without shoes, socks or brace. For radiographic measurement, standing anteroposterior radiographs of the whole spine without orthosis were taken. The coronal spinal length measurements were made on digital images at the PACS (Picture Archiving and Communications Systems, PACS) workstation. The intersection of catercorner in each vertebral body was defined as the centre of the vertebral body. Total length from radiographs along the line reaching the midpoint of both the superior and inferior endplates from T1 to L5, as well as the centre of each vertebral body in between was defined as the coronal spinal length (Fig. 1) [10, 11]. The calculation of HV and SGV were defined as the growth obtained from dividing the height increase by the time interval between two consecutive clinic visits at a minimal interval of 6-months [4, 12]: HV(SGV) = (Height (coronal spinal length)_n_ – Height (coronal spinal length)_n-1_) /(Time interval _n−(n−1)_). A growth velocity curve was then constructed for each patient. The age, at which the maximum growth velocity occurred, was designated as the peak height velocity (PHV) and peak spinal growth velocity (PSGV), respectively [12, 13].

The Cobb angle of the major curve of each subject was consecutively measured. Angle velocity (AV) was defined as increase in Cobb angle divided by the time interval between two consecutive clinic visits at a minimal interval of 6-months, expressed in angle degrees per year: AV = (Angle_n_-Angle_n-1_) /(Time interval _n−(n−1)_) [12]. The US Risser staging system was adopted [14]. For each patient, the visit with the highest AV value during the longitudinal follow-up together with the corresponding anthropometric and radiographic measurements, were selected for the final statistical analysis.

For the radiological measurements, a senior resident and a senior attending spine surgeon respectively measured the spinal length and Cobb angle twice at an interval of 1 week and the mean values were adapted for the analysis.

### Statistical methods

Data were statistically analyzed with the SPSS Statistics (v 17.0) software packages. The measured values were expressed as mean and standard deviation (SD). Descriptive statistics was performed to analyze patients’ demographics. For the intra- and inter-observer reliability analysis, the intraclass correlation coefficient (ICC) was calculated. The Chi-square test was used to compare the percentages of PHV and PSGV in the selected cases. The 2-tailed Pearson coefficients of correlation were calculated to assess the relationships between AV, SGV and other maturity indicators. Multiple linear regression analysis was performed to analyze the contributions of each maturity assessments to AV. Statistically significant difference was defined as *P* < 0.05.

## Results

A cohort of 62 IS girls were included in the study (Table 1). The average values of PHV and PSGV were 11.7 ± 4.6 cm/year (range, 4.0–20.5 cm/year) and 32.7 ± 15.4 mm/year (range, 8–93.3 mm/year), respectively, and the average timing of PHV and PSGV were 11.6 ± 1.4 years (range, 9.2–14.1 years) and 12.2 ± 2.3 years (range, 9.1–14.8 years), respectively. Table 2 showed the relatively good intra- and inter-observer reliabilities in the measurement of spine length.

**Table 1:** Summary of the 62 follow-up selected from 62 patients

	Mean (SD)	Range
Spine length (mm)	340.9 (20.0)	283–394
SGV (mm/year)	21.0 (13.4)	3–70
Standing height (cm)	155.2 (6.6)	132.5–165.5
HV (cm/year)	7.1 (4.9)	0.67–20.5
Cobb angle (°)	28.0 (6.0)	20–40
AV (°/year)	12.0 (10.6)	3.3–50

*AV* angle velocity, *SGV* spinal growth velocity, *HV* height velocity

**Table 2:** Intra- and inter-observer reliabilities analysis

Parameters	Intra-observer reliability (observer 1/observer 2)	Inter-observer reliability
Standing height	0.913/-	-
Spinal length	0.738/0.707	0.738
Cobb angle	0.938/0.946	0.833

### Chi-square test

**Fig. 2:**
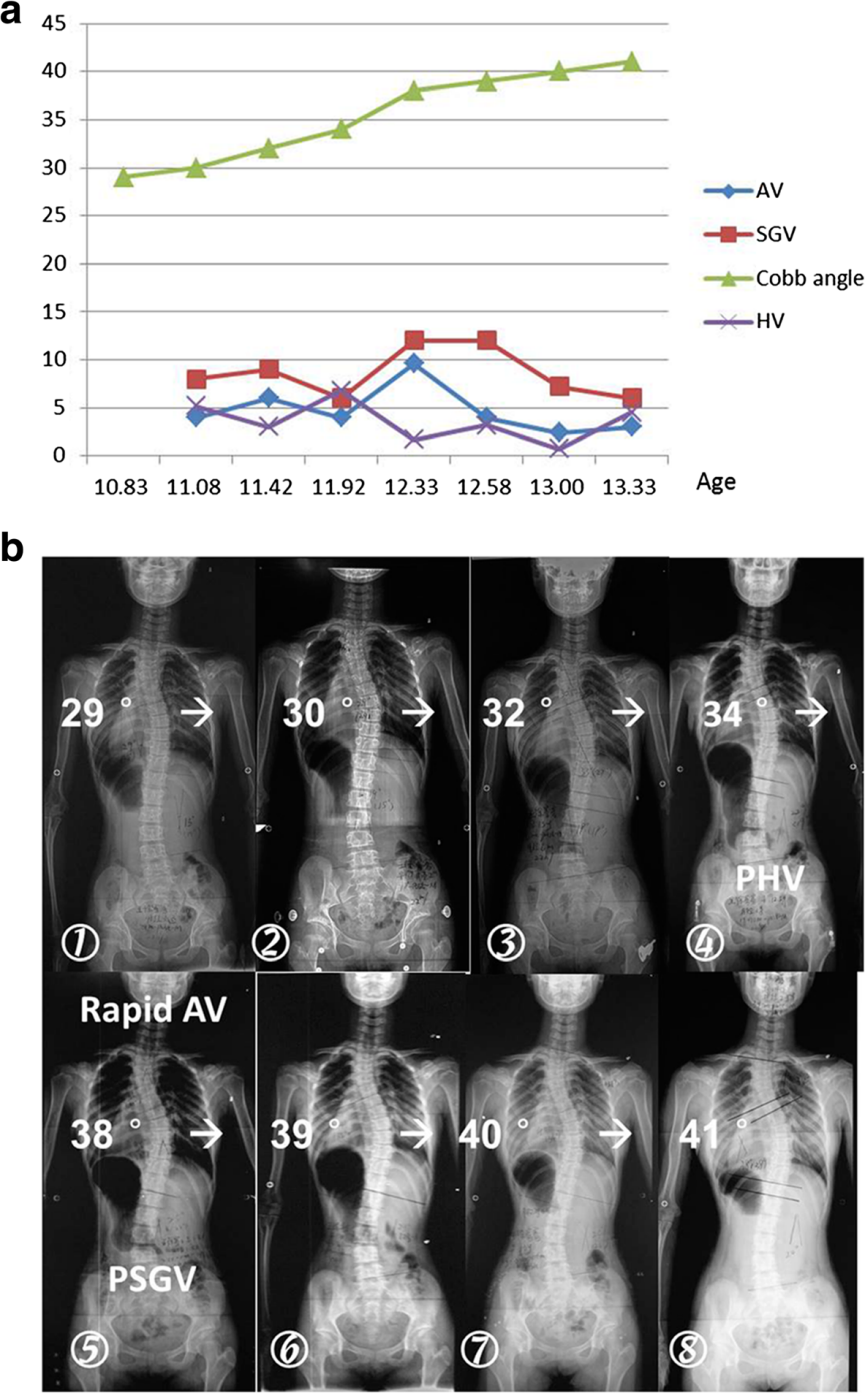
A demo case illustrating the influence of variations of growth velocity in relation to fluctuation of curve progressive velocity. **a**: Longitudinal curves of height velocity, spinal growth velocity, angle velocity and Cobb angle were constructed respectively. **a & b**: peak angle velocity occurred simultaneously with PSGV but not PHV

PHV was identified in 18 (29.0 %) and PSGV in 37 (59.7 %) cases, respectively. The Chi-square test revealed that PSGV contributed more to the occurrence of highest AV than PHV (*P* = 0.001). A demo case was shown in Fig. 2.

### Pearson correlation analysis

Pearson correlation analysis revealed significant correlation between SGV and HV (*r* = 0.394, *P* = 0.002). In addition, AV was significantly correlated with SGV (*r* = 0.454, *P* < 0.001) and HV (*r* = 0.280, *P* = 0.027), respectively. In contrast, no statistically significant correlation was detected between AV and other indicators of growth potential (Table 3).

**Table 3:** Correlation between AV/SGV and maturity indicators

	AV		SGV	
	r	P	r	P
Chronologic age	−0.068	0.599	−0.013	0.981
Risser sign	−0.155	0.229	−0.121	0.350
Spine length	−0.169	0.190	−0.028	0.831
SGV	0.454	<0.001	1	-
Standing height	−0.117	0.363	−0.242	0.059
HV	0.280	0.027	0.394	0.002
Cobb angle	0.189	0.142	0.037	0.773

Abbreviation: Refer to Table 1

### Multiple linear regression analysis

Interactions between AV and the maturity parameters including chronologic age, Risser sign, spinal length and standing height, together with Cobb angle of main curve were tested and excluded. The multiple linear regression analysis (Table 4) demonstrated that high AV was significantly predicted by higher SGV (B = 0.321, 95 % CI = 0.142–0.620, *P* = 0.007) rather than higher HV (B = 0.259, 95 % CI = −0.190–0.912, *P* = 0.362) (*R* = 0.467).

**Table 4:** Multiple linear regression analysis of AV

	B	95 % CI	P
Constant	3.393	−2.154–7.167	0.145
SGV	0.321	0.142–0.620	0.007
HV	0.259	−0.190–0.912	0.362

Abbreviation: Refer to Table 1

## Discussion

It is well known that the growth and curve progression in IS are closely interrelated [4, 15]. The most commonly used assessments of curve progression in IS included chronologic age, Risser sign, status of triradiate cartilage, menarche age, digital skeletal age, HV, secondary sexual characteristics, electromyography and hormonal levels [5, 10, 16–18]. A good and reliable understanding of the influence of peripubertal growth on the curve progression allows the surgeons to plan and prescribe the best strategy of treatment at the appropriate time. Sanders et al. [4] proved that the timing relative to peak height growth velocity was highly prognostic as it is significantly correlated with the curve acceleration phase. An increase of standing height more than 4 cm/year with curves more than 25° was significantly associated with increase in the angle velocity [19]. Escalada’s longitudinal study confirmed that PHV and peak angle velocity (PAV) took place simultaneously 1 year before menarche in progressive idiopathic scoliosis with bracing [12]. Despite being helpful as a first indication for curve acceleration phase, the predictive values of height velocity were partly downgraded by the existence of distal-to-proximal growth gradient in adolescents. The peak growth of distal body parts, for example, foot length or subischial leg length were found to precede the peak growth of more proximal body parts including the spine [7]. This growth gradient may introduce deviations when we use the height growth velocity as the first-line predictor of curve progression. Few studies, however, have been designated to investigate the influence of the spinal growth on curve progression.

In this study, serial longitudinal spine growth data measured from standing anteroposterior radiographs and the calculated SGV was documented. These longitudinal growth data did confirm the existence of a time gap between growth peak of body height and spinal length by an average of 0.6 years (timing of PHV and PSGV were 11.6 years and 12.2 years). In addition, correlation of maturity indicators with changes of AV was only found to be significant with SGV and HV, respectively.

A multiple linear regression model was created to further analyze the covariate effects of those which had significant association with AV in the crude analysis. Our results revealed that AV was significantly correlated with SGV rather than HV, indicating the major advantage of using SGV as an alternative to HV for predicting the change of AV. Similar results were reported by Ylikoski [20] that curve progression correlated with the spinal growth, and this correlation could be stronger with greater initial curve magnitude and with thoracic scoliosis. Wu et al. [21] reported that the correlation between spinal growth and curve progression was more prominent in the progressed group as compared with the stable group. This was in line with the fact that curve progression was determined by multiple factors, and spinal growth was one prominent but not the only decisive factor. A restricted cohort limited to progressive patients could help to highlight the accelerating effect of linear growth on curve progressive velocity in braced patients, as was confirmed in this study.

In addition, the Chi-square test showed the percentage of PSGV was much higher than that of PHV in the selected 62 follow-up with highest AV values of each patient, which further support the closer relationship between high AV and high SGV. Wever et al. [10] reported that the expected spinal growth at diagnosis was crucially important to further curve progression. In this retrospective study [10], a significantly greater curve progression rate in the rapid to moderate spine growth (≥10 mm per year) as compared with the smaller or no spine growth period (<10 mm per year) was observed. Based on these reports and results of our study, patients with pubertal spinal growth spurt undergoing bracing treatment should be monitored strictly and carefully for compliance and progress.

The underlying mechanism responsible for scoliosis progression during rapid spinal growth still remains controversial. It was hypothesized that the rapid asymmetric growth of the apical regions may result in exacerbated lateral deviation and curve progression according to the Hueter-Volkmann law [22]. Others have stressed the importance of the supportive musculoligamentous structures, which might fail to stabilize the growing spine because of the potential deficiency in the neuromuscular control system [23]. This theory was partially supported by the repeatedly confirmed association of susceptibility and curve progression of AIS with LBX1gene, which was expressed in the central nervous system and skeletal muscle and could play an important role in developmental processes [24]. Despite these plausible hypotheses, the detailed mechanism remains largely unknown.

Several common limitations shared by growth-related studies need to be addressed. Firstly, this study was limited by its retrospective nature and the relatively small sample size confined to girls with single and progressive curves. The conclusion might not be appropriate for complex curves in which the growth velocity might have a different influence on curve progression. The bracing treatment could significantly affect the curve progressive velocity. Despite the limitations, we do believe that analyzing the correlation between curve progression velocity and spinal growth velocity vs height velocity could provide useful information for the surgeon in designing the brace treatment strategy.

## Conclusions

This study has attempted to bridge the gap between spinal growth and curve progression. It was confirmed that variations of spinal growth velocity exerted more direct influence over changes in angle velocity (curve progressive velocity) as compared with height velocity in IS girls. In addition, high spinal growth velocity was found to predispose to higher incidence of rapid curve progression in the early pubertal period.

## Abbreviations

AV: Angle velocity
HV: Height velocity
IS: Idiopathic scoliosis
PHV: Peak height velocity
PSGV: Peak spinal growth velocity
SGV: Spinal growth velocity
